# Supplementary material for: Amplifying Coaches’ voices from the implementation of the MindSKILLZ youth mental health program in Kenya: A critical discourse analysis
Source: PLOS Glob Public Health. 2026 Sep 18;6(9):e0007336. doi: 10.1371/journal.pgph.0007336 (PMC13588515; doi:10.1371/journal.pgph.0007336)
Supplement: Supplementary file 1 [file pgph.0007336.s001.docx]

**LVCT Health & GRS**

**MindSKILLZ**

**Focus Group Discussion Guide for COACHES**

**Objective:**

- Understand effects of MindSKILLZ on mental health knowledge, attitudes, and care-seeking behaviours among Coaches

***Instructions to the facilitator:*** *Complete the following information prior to the interview. Read the introduction to the discussion participants.*

**Interview information:**

Date: ………………………………………………………………………………………………….

Venue/site: …………………………………………………………………………………………..

Start time: …………………………………………………………………………………………….

End time: ……………………………………………………………………………………………..

Facilitator name: …………………………………………………………………………………….

Co-facilitator/observer name: ………………………………………………………………………

Number of respondents, age & gender: …………………………………………………………..

**Instructions to facilitator:**

Introduce yourself to participants using the script below – it should be emphasized that participant names will not be used in any reporting, so they should feel free to speak their minds.

**Introduction**

First, let me introduce myself. “I am…, from XXX and I will be leading you through the discussion today.”

The **purpose** of this discussion is to collect information on your perspectives on some of the activities in the MindSKILLZ, the information in the curriculum, and its implementation. We will use this information to make improvements to the program.

We want to hear your open and honest views on the program - please remember that there are no ‘right’ or ‘wrong’ answers.

While we will be reporting on the information shared, no one’s name will be used in the notes.

I would like to record the session but only so that I can be sure that I am capturing everything you say. Is everyone okay with this?

You do not have to answer any questions that you do not want to answer, and you may stop participating at any time.

Please remember that we respect everyone’s views - it is a safe space to express your personal views. Please let me know if anything is not clear or you do not understand what I am asking.

**Do you have any questions before we get started?** [Note & answer any questions posed by participants]

**Thank you. Now, let’s get started with our discussion.**

| Topic | Main questions | Follow-ups/probes |
| --- | --- | --- |
| Overall views on program | Let’s talk about your experience as a Coach in the MindSKILLZ program.  How would you describe your experience as a Coach in the SKILLZ program? | What did you do as a Coach?  What are some of the things that you liked about being a Coach?  What are some of the things you disliked? |
| Views on MindSKILLZ activities | Now let’s talk more specifically about the activities in MindSKILLZ. [*Can remind them about some sessions/activities*]  What information and skills are participants supposed to learn in MindSKILLZ? | Probe: What are they meant to learn about mental health, stress, etc?  What are some of the key messages that were important to deliver during these activities? |
|  | How well do you think participants learned about mental health and wellbeing? | Do you have any examples to share?  What activities/sessions were difficult for them to understand, if any? |
|  | How did you feel while facilitating the MindSKILLZ activities? | Probe: did it make you feel upset or worried? Hopeful/optimistic?  Can you explain what made you feel this way? |
|  | What are some things you liked about the sessions? What are some of the things that you disliked? | What activities were difficult to implement, if any?  Did you ever modify or skip any of these activities? Why? |
| Views on MindSKILLZ implementation | What challenges did you face in implementing the MindSKILLZ activities? | How did you address these challenges?  What would help you address these challenges in the future? |
|  | How well did the MindSKILLZ training prepare you for facilitating these activities? | Did participants have any questions that you weren’t able to answer?  What additional training, if any, would help you improve facilitation of these activities? |
| Impact of MindSKILLZ on Coaches | Now let’s talk more about the impact of MindSKILLZ on your knowledge, attitudes, and skills.  Did you personally learn about mental health through your work as a Coach?  Compared to before you were trained and facilitated MindSKILLZ, have you changed the way you act or think? | Can you tell us more about what you learned?  Can you give any examples?  Do you use any knowledge or skills you gained outside of MindSKILLZ? |
| Recommendations for Improvement & Closure | Now we are reaching the end of the discussion.  Thinking about the MindSKILLZ program, what recommendations do you have to make it better?  Is there anything else you’d like to share with us? (Further comments, questions, etc.)  **Thank you!** | Probe: information, activities, duration, etc. |
