# Supplementary material for: Amplifying Coaches’ voices from the implementation of the MindSKILLZ youth mental health program in Kenya: A critical discourse analysis
Source: PLOS Glob Public Health. 2026 Sep 18;6(9):e0007336. doi: 10.1371/journal.pgph.0007336 (PMC13588515; doi:10.1371/journal.pgph.0007336)
Supplement: Supplementary file 4 [file pgph.0007336.s007.docx]

| **Section** | **Reviewer’s Comment** | **Author’s Response** | **Additional Notes** |
| --- | --- | --- | --- |
| **Feedback from Academic Editor** | | | |
| Participant Consent | Please provide additional details regarding participant consent. In the ethics statement in the Methods and online submission information, please ensure that you have specified (1) whether consent was informed and (2) what type you obtained (for instance, written or verbal, and if verbal, how it was documented and witnessed). If your study included minors, state whether you obtained consent from parents or guardians. If the need for consent was waived by the ethics committee, please include this information. | This analysis focused on Coaches of MindSKILLZ who were all adults (aged 18+ years). We clarified in the Ethical Considerations section that we obtained written informed consent prior to data collection. | N/A |
| Use of data from medical records or archived samples | If you are reporting a retrospective study of medical records or archived samples, please ensure that you have discussed whether all data were fully anonymized before you accessed them and/or whether the IRB or ethics committee waived the requirement for informed consent. If patients provided informed written consent to have data from their medical records used in research, please include this information. | Our study targeted collection of qualitative data using face to face interviews and personal journaled stories. No medical records or archived samples were used. | N/A |
| Manuscript Upload | We ask that a manuscript source file is provided at Revision. Please upload your manuscript file as a .doc, .docx, .rtf or .tex. | This has been done. | N/A |
| Recommendation to cite specific published work | If the reviewer comments include a recommendation to cite specific previously published works, please review and evaluate these publications to determine whether they are relevant and should be cited. There is no requirement to cite these works unless the editor has indicated otherwise. | The reviewer did not recommend citing any specific published work. | N/A |
|  | Do you want your identity to be public for this peer review? If you choose “no”, your identity will remain anonymous but your review may still be made public. | Yes | N/A |
| **Comments from Reviewer #1** | | | |
| Overall | The study rationale is unclear. Authors state: We were interested in understanding their experiences implementing a mental health intervention for young people during a pilot study, including any program impacts on Coaches themselves and on youth participants. Why was this done? To inform future programming? | This study was done to identify implementation successes and challenges, improve use of language in developing materials for program implementation, strengthen institutional guidelines for learning materials and generate evidence to inform future program delivery, adaptation and scale-up. | We have revised the study rationale in the last sentence of the Introduction section  Page 4 |
|  | How does this approach compare to other approaches of using youth cadre to deliver mental health initiatives in this region e.g., Youth Friendship Bench. See: Beji-Chauke R, Simms V, Abas M et al. A cluster-randomized trial of interventions for adolescent mental disorders in Zimbabwe. BMC Psychiatry. 2025;25(1):673. doi: 10.1186/s12888-025-06755-x. | The two approaches are similar as both interventions engage older peer mentors to reach adolescents with mental health messages. However, the MindSKILLZ approach varies from the Youth Friendship Bench intervention in that the MindSKILLZ engages young peer mentors (Coaches) within the community to deliver the intervention and the Coaches are older than the adolescent participants 10-14 years. The Youth Friendship Bench, on the other hand, engages University student as peer mentors to support adolescents ages 16-19 years. | Included this perspective in the introduction section  Page 4 |
|  | The manuscript is marred by numerous typographical and grammatical errors. This affects the readability of this work. Careful proof reading and editing is needed. | Thank you very much for this observation. We have reviewed the entire manuscript and made corrections appropriately. | We have made edits throughout the manuscript |
|  | Title could include study type e.g. Amplifying Coaches’ Voices from the Implementation of the MindSKILLZ Youth Mental Health Program in Kenya: A Critical Discourse Analysis | Thanks, we have revised the title. The new title reads: *Amplifying Coaches’ Voices from the Implementation of the MindSKILLZ Youth Mental Health Program in Kenya: A Critical Discourse Analysis* | Page 1 |
| Abstract | Abstract should state study dates | We have included the study dates. | Page 1 |
| Introduction | HIV/AIDS remains a leading cause of death among adolescents in SSA – include citation. | Citation included. | Citation no 3 Page 3 |
|  | SSA also has the highest rate of adolescent’s pregnancies…- SSA also has the highest rate[s] of [adolescent] | Corrected. | Page 3 |
|  | Evidence shows that interventions aimed at promoting mental wellbeing of adolescents and preventing mental health outcomes… Check this - mental health outcomes can be either positive or negative. Positive mental health outcomes include for example, self-worth. | We have revised this statement, replaced ‘mental health outcomes’ with ‘mental health concerns’ | Page 3 |
|  | In Kenya, many people attribute mental illness to spiritual or supernatural caused of mental illness – check this sentence. | Thank you. We have revised this statement to address this. | Page 3 |
|  | This lack of understanding contributes significantly to stigma surrounding mental health issues (7). Unsure if this is a lack of understanding as mental illness could be due to many causes. Also, do authors intend to say mental illness or simply mental health problems. From which positionality are authors stating this? This is what people believe and this belief should be respected as opposed to being dismissed as ‘nonsense’. | Thanks a lot for this observation. We have revised the sentence. | Page 3 |
|  | LVCT Health is a Kenyan non-governmental organization that implement [implements] health programs and scale [scales]?… | We have made these corrections. | Page 4 |
|  | By designing and implementing innovative health programs in the areas of HIV, sexual and reproductive health, maternal and child health, sexual violence prevention, mental health, and health systems strengthening (9) GRS is an adolescent health organization using the power of soccer to educate, inspire, and mobilize young people to live healthier lives across over 60 countries (10)(cite). Is this one sentence? See (cite) at end of sentence. | Thank you. We have deleted the ‘(cite)’ in this sentence. | Page 4 |
| Material, Methods and Approaches [Materials and Methods] | Material, Methods and Approaches [Materials and Methods] | Thank you this has been actioned and now reads ‘(Materials and Methods)’ |  |
|  | Please include study guide as supplementary (S1) file | Thank you. We have uploaded this as a supplementary file | N/A |
|  | and adolescents are predisposed to an environment that adversely affects their psychosocial health. Please say more about the environment. | Thank you. This has been included. Literature shows that adolescents living in peri-urban areas like those where this study took place are pre-disposed to conditions such as parental absence due to work, conflict at home, overcrowded living conditions and insecurity, coupled with low access to mental health services | Study Setting and Pilot Overview section.  Page 4 |
|  | As part of the ITS design participant completed [participants]? | Thank you for the correction. Adjusted. | P5 |
|  | All 30 Coaches were invited to participate in FGDs. Of these, 23 Coaches agreed to participate in 3 FGDs, with an average of 7 participants each.  Why did the other 7 refuse to participate? | The 7 Coaches were not available on the day FGDs were taking place. | N/A |
|  | FGD [FGDs] together with Coaches’ personal stories | Thanks, we have corrected the typos. | Page 6 |
|  | They met regularly with two other researchers in a team of four to discuss the coding process, compare codes, and adjust approaches where needed to enhance consistency in the application of codes.  Were there any disagreements? If so, how were these resolved? | We have included this information:  ‘*In instances where there were disagreements between coders, open discussions were held during which each coder provided justification for the inclusion of the specific codes. The coders together with the senior researchers reviewed the disputed segment to reach to a consensus*.’ | Data Analyses section  Page 6 |
|  | …how this might have influenced the research process and the findings, [findings.] | Thank you, this has been corrected | Reflexivity section  Page 7 |
| Ethical Consideration[s] | Ethical Consideration[s] | Corrected | Page 8 |
|  | It also received approval by the [from the] | Corrected | Page 8 |
| Results | Text (Micro Level) This analysis focused on the textual and linguistic features of Coaches’ statements, all of which represented success stories relating to the program’s impact and implementation. This is repetitive. | We have deleted this. | Page 9 |
|  | participants living with disability [participants with disability] | Thank you. We revised this in this context and throughout the manuscript | Page 13 |
|  | adolescents living with disabilities [adolescents with disabilities] | Corrected | Page 13 |
|  | institution way [institutional]? | Corrected | Page 15 |
|  | Coaches suggested that the language translated in the program documents and pre and post survey that were written in English Coaches feedback on program documents and pre and post survey revealed challenges particularly related to social power imbalances. Check this. | This has been revised. Thank you. | Page 15 |
|  | set -ups [set-ups] also spelt setups | Corrected | Page 16 |
|  | approaches that enhances [enhance] | Corrected | Page 16 |
|  | This conflict with community realities highlighting institutional discourse failure in the alignment of cultural contexts. | Corrected | Page 17 |
| Discussion | • A qualitative study conducted in Zambia results highlights how facilitators also experienced personal growth whilst implementing a youth led intervention (21). Check this. | Corrected | Page 17 |
|  | In addition to improvements observed among Coaches our findings also revealed [suggested]? | Corrected | Page 17 |
|  | especially with young people living with disability [especially with young people with disability] | Corrected | Page 18 |
|  | have excluded people living with disabilities [people with disabilities] | Corrected | Page 18 |
|  | Health inequities for children is an indicator of scarce health intervention[s]…Lower Middle-Income Countries [Low to Middle-Income Countries]? | Thank you, we have revised this to:  ‘*Low and Middle-Income Countries (LMICs).’* | Page 18 |
|  | Studies emphasizes [emphasize] | Corrected | Page 18 |
|  | According to the Kenya Community Health Policy 2020–2030, CHPs are selected within the localities that they reside and who are able to speak the local language (25). Check this. | We have adjusted this sentence. | Page 18 |
|  | Limitations section could also focus on strengths e.g., Study strengths and limitations. | We have revised the heading of this section to read:  *‘Study Strengths and limitations’* and provided information on study strengths. | Page 19 |
| **Comments from Reviewer #2** | | | |
| Overall | Overall, I believe that this is an interesting paper highlighting the important gap by focusing on the experiences of implementers (i.e., youth Coaches) rather than only participants. The paper illustrates how the perspectives of Coaches directly shape the delivery and effectiveness of youth mental health interventions. | Thank you very much for this positive feedback. We hope that this paper will shape future implementation and effectiveness of similar mental health interventions targeting young people. | N/A |
| Introduction | ‘poor reproductive and sexual health outcomes, and troubled parent/child relationships (1).’ Consider rewriting it to ‘Poor sexual and reproductive health outcomes’ | Corrected | Page 3 |
|  | Are these rates referring to prevalence in SSA or different countries? Please make this clear | We have clarified the statistics for SSA versus global statistics. | Page 3 |
|  | ‘ref’ – please kindly add the reference | We added the reference. | Reference no 7  Page 3 |
|  | The inclusion of Kenya would benefit from clearer integration into the narrative. It may be helpful to introduce the focus on Kenya more explicitly and provide a rationale for its inclusion in relation to the study. This would strengthen the argument regarding the implementation of the intervention and enhance the overall relevance of the study within the Kenyan context. | Thank you. We have included the adolescent mental health perspective from Kenya to strengthen the introduction. | Page 3 |
|  | reads like it was forgotten and included at the last minute. Please find a way to introduce Kenya and explain why focusing on Kenya as a whole is relevant to the study. It would support the argument that is being made about the implementation of the intervention and the relevance of the study in Kenya. | We have improved the section. | Page 3 |
|  | You can also highlight the mental health scope in Kenya before discussing the lack of support. This will strengthen the argument you are making. | This has been actioned as suggested. | Page 3 |
|  | If data is available, you can describe the ratio of individuals with mental health conditions to available health personnel in Kenya. This would help to contextualise the capacity of the health system. | Thank you. We have provided some statistics on adolescent mental health in Kenya. | Page 3 |
|  | If possible, please spell out LVCT in full at first mention before using the acronym throughout the paper | LVCT Health (formerly Liverpool VCT) is a registered NGO in Kenya therefore not an acronym. | N/A |
|  | Add a full stop after reference 9 | Corrected | Page 3 |
|  | After reference 10, you wrote ‘cite’ – not sure if you forgot to remove this or wanted to include another citation. Please update accordingly. | Corrected | Page 4 |
| Methodology | Provide references in paragraph 1 in the last two sentences. | Corrected | Page 5 |
|  | What is GRS? Please spell out the acronym to ensure clarity. | GRS stands for Grassroot Soccer Inc. We spelt it out in the Introduction section. | Paragraph 4 of the Introduction section (Page 4). |
|  | Briefly explain the ‘necessary knowledge and skills' that the coaches require to deliver the sessions. | We have included this information.  ‘*The Coaches were trained on ways to support young people in expressing and managing their emotions, coping with stress, seeking support, and creating safe spaces for meaningful interactions with young people.*’ | Page 5 |
|  | Please provide more detail on the recruitment strategy described in paragraph 2, including how participants were identified and the sampling approach employed by the coaches. | We used school registers and lists from the communities to randomly sample adolescent participants. Only those who provided parental consent and assent were enrolled into the program. | Paragraph 3 under ‘Study Setting and Pilot Overview’ Page 5 |
|  | Please briefly describe the content of the ‘pre’ and ‘post’ surveys mentioned in paragraph 2, including the key domains or topics covered. | This has been included;  *‘pre and post surveys which measured mental health knowledge and attitudes as well as mental health-seeking behavior).’* | Paragraph 4 under ‘Study Setting and Pilot Overview’ Page 6 |
|  | Please clarify what is meant by a ‘participant in need’. Additionally, indicate whether any follow-up procedures were conducted to assess whether participants accessed the recommended services, and if so, which services were utilised (paragraph 3) | Thanks a lot. We have clarified this: participants in need of mental health support.  We have also indicated that follow-ups were made to ensure such participants received necessary mental health support. | Paragraph 3 of ‘Study Setting and Pilot Overview’  Page 6 |
|  | Were the coaches compensated? | Yes, they received monthly compensation of Ksh 10,000 for facilitating four 2-hour sessions per month | ‘Paragraph 3 of ‘Study Setting and Pilot Overview’  Page 6 |
| Findings | Kindly describe the population (how many coaches, age, education status, etc) | We have included a narrative and a table describing the characteristics of study participants. | Characteristics of Study Participants  Page 9 |
|  | b) Improved self-esteem and academic performance of the youth program participants.  Based on the quote provided, I don’t believe it captures what you described as ‘This observation connotes how self-esteem can have a ripple effect on academic outcomes.’ I can see the improved self-esteem and academic performance, but from the provided quotes, I can’t see how one informs the other or the link that you infer in the paragraph after the quote on self-esteem. | We have revised the paragraph to capture the two independent unintended outcomes: self-esteem and academic performance. | Page 9 |
|  | Throughout the program, her self-esteem improved quite significantly to a point that she started talking about herself freely and interacting with the rest  o Was this interaction observed in class, too or in the program? | We have included part of the quote that describes feedback from school. | Page 10 |
|  | b) Social Power Relations  Rewrite the first sentence; it is not clear. | Corrected | Page 14 |
|  | If possible, please provide examples of the specific terminology being referred to. | Thank you, the terms are:  *‘Resilience, sympathy and empath.’* | Page 14 |
|  | Additionally, it may be useful to clarify whether the challenge was primarily related to the terminology itself or to how these concepts were explained during translation. Based on the quote provided, it appears that some participants may have interpreted the sessions as being targeted at individuals with mental health disorders, rather than as an opportunity to learn about mental health more broadly. Clarifying this distinction could strengthen the interpretation of participant dropout. | The challenge was primarily based on how concepts were translated to Kiswahili. Mental health concepts lack direct Kiswahili translations, meaning a translator has to use a phrase or a long sentence to describe a single mental health concept. This may lead to loss of intended meaning. | P14 |
| Discussion | Rewrite the 6th sentence that speaks about the results from Zambia. | Corrected | Page 17 |
|  | ‘Most mental health programs have excluded people living with disabilities, despite the fact that they face heightened risks of mental health challenges, often worsened by their disabilities and the stigma associated with them.’ - Reference this statement. | We have included the reference. | Page 17 |
|  | Rewrite the first part of the sentence following the discussion about health inequities in children. | Corrected | Page 18 |
|  | For the last sentence in paragraph 3, maybe you can provide examples of what the coaches employed. | We have provided examples of approaches Coaches employed to reach participants with disabilities. | Page 18 |
|  | If possible, maybe describe why a decision was made to exclude the CHPs from recruiting. | CHPs were not involved in the pilot because the program sought to engage young people who resonated with adolescents. | An explanation is provided in the paragraph 3 of ‘Study Setting and Pilot Overview’ (Page 5)  Page 18 |
|  | According to the Kenya Community Health Policy 2020–2030, CHPs are selected within the localities that they reside and who are able to speak the local language (25)’ This sentence feels a bit misplaced; maybe move it up or elaborate more in terms of the importance of CHPs according to the health policy to support your argument. | Thank you. We also noted that the sentence was misplaced. We have since placed it within its appropriate paradigm. | Page 18 |
