# Supplementary material for: Amplifying Coaches’ voices from the implementation of the MindSKILLZ youth mental health program in Kenya: A critical discourse analysis
Source: PLOS Glob Public Health. 2026 Sep 18;6(9):e0007336. doi: 10.1371/journal.pgph.0007336 (PMC13588515; doi:10.1371/journal.pgph.0007336)
Supplement: Supplementary file 5 [file pgph.0007336.s005.docx]

**Amplifying Coaches’ Voices from the Implementation of the MindSKILLZ Youth Mental Health Program in Kenya: A Critical Discourse Analysis**

**Authors**: Benard Nyauchi^1^, Mercy Kihiu^2^, Robert Kimathi^1^, Devyn E.Z. Lee^2^, Antony Chazara^2^, Elizabeth Okoth^2^, Stella Waruinge^3^, Celina Kithinji^4^, Katherine G. Merrill^5^, Andy Dallos^2^, Ilana Cohen^2^, Lilian Otiso^1^

**Author Affiliations**

^1^LVCT Health, Kenya

^2^Grassroot Soccer, Inc., Hanover NH, USA

^4^County Government of Nairobi, Kenya

^5^County Government of Mombasa, Kenya

^6^Center for Dissemination and Implementation Science, University of Illinois Chicago, USA

**Abstract**

Young implementers bring valuable insights that can help inform and create effective mental health support systems and interventions tailored to their needs and unique experiences. However, their perspectives are often overlooked. This qualitative study analysed discourses from community role models, referred to as MindSKILLZ Coaches. We hosted five focus group discussions and collected 30 personal stories journaled by MindSKILLZ Coaches in Nairobi and Mombasa counties between August 2023 and March 2024. We were interested in understanding their experiences implementing a mental health intervention for young people during a pilot study, including any perceived program impacts on Coaches themselves and youth participants.This was done to identify implementation successes and challenges, improve use of language in developing materials for program implementation, strengthen institutional guidelines for learning materials and generate evidence to inform future program delivery, adaptation and scale-up. Data were analysed using Fairclough’s Model for Critical Discourse Analysis to examine three interconnected discourses: 1) *textual practice* (micro level), 2) *discursive practice* (meso level), and 3) *social practice* (macro level). Across these levels, we looked at how discourse contributes to the maintenance of social power relations, ideologies, and institutional practices. Findings revealed that the MindSKILLZ program positively impacted the Coaches and youth participants by improving self-esteem, use of creative approaches to reach persons with disability, and improvement of academic performance, all of which facilitated successful program implementation overall. Institutional practices (e.g. assumptions of uniform cultural practices among participants), structural power imbalances (e.g. limitations on Coaches’ roles in program implementation) and social relations (e.g. Coaches simplified language reinforcing their authority) limited the program’s effectiveness. Amplifying the Coaches’ voices enhances adoption of a grassroots perspective, which can challenge unequal power dynamics. The use of grassroots insights can aid in co-creating more equitable and effective mental health interventions.

**Introduction**

Adolescents aged 10-19 years in sub-Saharan Africa (SSA) constitute 23% of the region’s population, a figure projected to rise from the current 256 million to 435 million by 2050 (1). As the adolescent population in this sub-continent grows, young people continue to face health problems and socio-economic challenges, such as HIV/AIDS, teenage pregnancies, and food insecurities, which heighten their risk of mental health problems (2). HIV/AIDS remains a leading cause of death among adolescents in SSA (3). SSA also has the highest rates of adolescent pregnancies in the world, with 109 births per 1,000 adolescent girls (aged 10–19 years) compared to the global average of 42 births per 1,000 adolescents (4). The majority of adolescents in this region live in extreme poverty (5), a situation exacerbated by rapid urbanization which has led to increased urban slums. This environment fosters a rise in mental health challenges which may lead to adolescents’ poor academic performance, aggressive behaviors, poor sexual and reproductive health outcomes, and troubled parent-child relationships (1). Over time, these difficulties can worsen, resulting in severe impairments like substance abuse, depression, and suicide attempts in adulthood (6).

Adolescence is a critical time for prevention and treatment of mental ill health, as 63% of global mental disorders occur by the age of 25 years (7,8). Evidence shows that interventions aimed at promoting mental wellbeing of adolescents and preventing mental health concerns can improve mental health literacy and emotional skills, which in turn reduces symptoms of anxiety and depression, thus improving individual health outcomes overall (9). However, countries in SSA still face the challenge of lack of robust data on adolescent mental health. The few studies that exist report high prevalence of mental health issues, including a 27% for depression, 30% for anxiety disorders, and 40% for emotional and behavioral problems (10).

In Kenya, young people face mental health challenges, including peer and social pressures, drug and substance use, absence of parental or caregiver support, inadequate copying mechanisms and limited knowledge on steps to take towards mental health prevention (11). Service provider challenges include poor attitudes towards mental health issues, lack of confidentiality in healthcare settings, and limited promotive and preventive strategies to avert mental health issues (12). Prevalence data for adolescent mental conditions in Kenya is scarce, but a recent study with high school youth found high levels of depression and anxiety symptoms, with about 46% exceeding clinical cut-off for depression, and about 38% exceeding the clinical cut-off for anxiety (13,14). Adolescents with mental health challenges receive sub-optimal support as the country faces a severe shortage of mental health specialists, with only 150 psychiatrists and 12 neurologists who are mostly located in the urban settings of Nairobi, Kisumu, and Mombasa and available in private rather than public settings (10). Furthermore, many people attribute mental illness to spiritual or supernatural causes, a belief that has potential to contribute significantly to stigma surrounding mental health issues (9).

To tackle some of these challenges, LVCT Health and Grassroot Soccer (GRS) partnered with the County Governments of Nairobi and Mombasa to pilot the MindSKILLZ program for the first time in Kenya. LVCT Health is a Kenyan non-governmental organization that implements health programs and scales them in Kenya and beyond (15). GRS is an adolescent health organization that uses the power of soccer to educate, inspire, and mobilize young people to live healthier lives across over 60 countries (16). MindSKILLZ is a sport-based mental health promotion program for adolescents seeking to improve their mental health knowledge, skills, and attitudes, and decrease stigma around mental health. MindSKILLZ utilizes the GRS’s sport- and play-based approach to learning about critical health topics (17). In this approach, young adult mentors in the community, known as Coaches, are recruited and trained on youth facilitation skills and program content to deliver the sessions and act as positive role models for adolescent beneficiaries (11).

In recent years, youth engagement has emerged as a guiding principle for redesigning and shaping youth mental health interventions (18). However, young adult implementers are often overlooked, yet they are a key driving force to influencing change. A study conducted in Zimbabwe established that engaging university students as peer mentors to support adolescent mental health had the potential to connect them with same-age peers to enhance accessibility of mental health services (19). Moreover, engaging young people as change agents builds confidence and recognizes youth’s rights for agency and power in shaping mental health interventions (20). This study uses Norman Fairclough’s (1989,1995) three-dimensional model of Critical Discourse Analysis (CDA), which is structured to examine discourses at three levels: micro, meso, and macro levels (21). We sought to analyse Coaches’ experiences implementing MindSKILLZ and personal stories by examining how these discourses reinforce or challenge social power imbalances and institutional practices in mental health programming. Our goals were to identify implementation successes and challenges, improve the use of language in developing materials for program implementation, strengthen institutional guidelines for learning materials, and generate evidence to inform future program delivery, adaptation, and scale-up.

**Materials and Methods**

**Study Setting and Pilot Overview**

A pilot of MindSKILLZ was carried out in the Westlands and Ruaraka sub-counties in Nairobi County and in Kisauni, Jomvu, Likoni, and Changamwe sub-counties in Mombasa County between August 2023 and March 2024. These locations were selected based on their low socio-economic situation, existence of sub-county mental and school health support systems, and the potential of recruiting adolescents aged 10-14 years for the pilot. Adolescents living in these localities often experience parental absence due to work, conflict at home, overcrowded living conditions and insecurity, coupled with low access to mental health services which predisposes them to depression and anxiety disorders (22).

The pilot used an interrupted time series (ITS) design, which sought to assess intervention effects on mental health outcomes particularly mental wellbeing and depressive symptoms (11). Sessions were delivered by trained Coaches in both Nairobi and Mombasa counties. These were young people perceived to be role models, residing in the respective study sites, who would be responsible for delivering the MindSKILLZ program to adolescent participants. The recruitment aimed at identifying dedicated individuals between the ages of 19 and 24 years who were passionate about youth or community development and possessed basic facilitation skills, a high school education, and a police clearance certificate. Partnerships with existing youth networks were leveraged to reach potential candidates, and the opportunity was widely advertised. Following the shortlisting process, interviews were conducted to assess candidates' suitability, and those selected underwent an orientation on the MindSKILLZ program. The orientation provided an overview of program objectives, expectations, and the role of SKILLZ Coaches in delivering mental health sessions to adolescents.

The Coaches were taken through a 5-day training facilitated by experienced GRS and LVCT Health trainers, equipping them with the necessary knowledge and skills to deliver MindSKILLZ sessions effectively. The Coaches were trained on ways to support young people in expressing and managing their emotions, coping with stress, seeking support, and creating safe spaces for meaningful interactions with young people. Adolescent participants were recruited through randomization from school registers and community lists by the trained Coaches. The adolescent participants were selected from the community and school set-ups after parental consent and assent were obtained. Whereas typically LVCT Health engages community health promoters (CHPs) to recruit participants, CHPs were not involved in the pilot because the program sought to engage young people who resonated with adolescents in their respective communities. In Nairobi, 18 Coaches were involved in the implementation of the program in the two sub-counties, reaching 1,935 participants. In Mombasa, 12 Coaches implemented MindSKILLZ and reached a total of 1,764 participants. Each Coach received a monthly compensation of Ksh 10,000 (~US$XXX) for facilitating four 2-hour sessions per month.

The MindSKILLZ program, which was co-designed with youth, includes 12 sessions, covering topics ranging from basic mental health knowledge to positive coping skills. Coaches delivered the in-person sessions, and a printed comic book-style magazine was provided to participants to reinforce content from the in-person sessions. Further description of the development of MindSKILLZ is found elsewhere (11). The program further aimed to connect young people to mental health support services. Coaches referred participants in need of mental health support to LVCT’s one2one platform, a youth hotline and digital health platform that includes chatbots, peer mentors, and professional counsellors. The Coaches made follow-ups to ascertain that such participants accessed and utilised the recommended services. As part of the ITS design, participants completed brief surveys immediately before and after the intervention (i.e., “pre” and “post” surveys) which measured mental health knowledge, attitudes as well as mental health-seeking behaviour.

**Study Design**

This study was conducted as a qualitative sub-study within the larger ITS pilot. We drew on focus group discussions (FGDs) and Coaches’ journaling of personal stories to capture the perspectives of Coaches implementing MindSKILLZ during the pilot.

**Data Collection**

A team of 10 trained research assistants with requisite professional and academic qualifications carried out qualitative data collection. All 30 Coaches were invited to participate in FGDs. Of these, 23 Coaches participated in 3 FGDs, with an average of 7 participants each. The 7 Coaches who did not join the FGDs were unavailable on that day. Two of the FGDs were held in Nairobi and one in Mombasa. Coaches who agreed to participate were guided through an informed consent process by the research assistants before their involvement in the FGDs. Semi-structured guides prompted Coaches to share detailed narratives about their experiences, challenges and perceived outcomes of the MindSKILLZ program. The discussions were led by a skilled moderator and supported by a note-taker, and were conducted in both Swahili and English languages. The FGDs lasted between 90-120 minutes each and were audio-recorded. In addition to the FGDs, the study analysed personal stories from 16 Coaches journaled during implementation of MindSKILLZ program to further understand Coaches’ experiences in implementing the intervention activities.

**Reflexivity**

A reflexivity session was held with the broader research team to reflect their positionality. The process was continuous and collaborative and it allowed the researchers to self-consciously critique, appraise and evaluate how their subjectivity and context likely influenced the research process (24). For example, the broader research team consisting of 12 individuals (10 research assistants and 2 research leads) examined their own roles and biases and documented how this might have influenced the research process and the findings.

**Data Analyses**

FGD recordings were transcribed verbatim in Microsoft Word by a team of experienced research assistants. FGDs together with Coaches’ personal stories were coded with the support of NVivo™ 12 (QSR International Pty Ltd: Doncaster, Australia). An initial deductive coding framework, based on the study objectives, was developed *a priori* by the research team. This framework was then expanded through inductive coding to capture emerging themes identified during analyses. Coding was conducted independently by two researchers, who met regularly with two other researchers to discuss the coding process, compare codes, and adjust approaches where needed to enhance consistency in the application of codes. In instances where there were disagreements between coders, open discussions were held during which each coder provided justification for the inclusion of the specific codes. The coders, together with the senior researchers, reviewed the disputed segments to reach a consensus. Codes were grouped and analysed according to three levels of Fairclough’s Model for Critical Discourse Analysis (15):

- Textual Practice (Micro Level): This level centred on the language used, focusing on linguistic features such as vocabulary, grammar, and rhetorical devices through Coaches’ descriptions of their experiences during implementation of the program.
- Discursive Practice (Meso Level): This level explored how Coaches’ discourses were produced, distributed, and consumed within MindSKILLZ program implementation. Analyses focused on how these narratives were shaped by the intervention and how they were received by various audiences.
- Social Practice (Macro Level): This level examined the broader social and cultural structures that shaped and/or were shaped by discourse. We looked at how Coaches’ discourse contributed to the maintenance of social power relations, ideologies, and institutional practices.

Data were triangulated across the data collection methods (i.e., FGDs and Coaches’ journals) to facilitate comparisons and generate a deeper understanding of the findings (23).

**Ethical Considerations**

This study received ethical approval from Amref Health Africa’s Ethics and Scientific Review Committee (ESRC) - ESCRC P1389/2023, as well as approval from the National Commission for Science, Technology, and Innovation (NACOSTI) and from the County Department of Health research committees in Nairobi and Mombasa. All participants provided written informed consents prior to participation in the data collection process.

**Findings**

We proceed to present findings according to the three levels of Fairclough’s CDA model: 1) textual practice (Micro Level); 2) discursive practice (Meso Level); and 3) social practice (Macro Level). To ensure anonymity while conveying the Coaches’ voices, pseudonyms were used for the attribution of the quotes given by the Coaches throughout this section.

**Characteristics of Study Participants**

A total of 23 Coaches participated in this study, of whom two-thirds were female. More than half of the participants (60.9%) were aged below 25 years, with a mean age of 24.3 years and the median age of 24.1 years [IQR:23.0-25.5]. Additionally, the majority of participants (87.0%) were single. In terms of education attainment, 73.9% had completed post-secondary education.

| **Demographic Characteristic** | | **Frequency** | **%** |
| --- | --- | --- | --- |
| **Age of participant** | |  |  |
|  | 21 years | 1 | 4.3 |
|  | 22 years | 3 | 13.0 |
|  | 23 years | 4 | 17.4 |
|  | 24 years | 6 | 26.1 |
|  | 25 years | 4 | 17.4 |
|  | 26 years | 2 | 8.7 |
|  | 27 years | 1 | 4.3 |
|  | 28 years | 2 | 8.7 |
| **Sex of participant** | |  |  |
|  | Male | 10 | 43.5 |
|  | Female | 13 | 56.5 |
| **Marital status** | |  |  |
|  | Single | 20 | 87.0 |
|  | Married | 3 | 13.0 |
| **Education level** | |  |  |
|  | Secondary | 1 | 4.3 |
|  | Certificate | 2 | 8.7 |
|  | Diploma | 11 | 47.8 |
|  | Degree | 6 | 26.1 |
| Total | | 23 | 100.0 |

**Textual Practice (Micro Level)**

a) **Empowerment of Coaches**

The language and wording used by Coaches across Nairobi and Mombasa suggested that Coaches were empowered by the skills they received while implementing the MindSKILLZ program. Coach John in Nairobi reflected:

*Newfound skills have empowered me to navigate the complexities of the mental health world with greater confidence and clarity*. (Coach in Nairobi).

The statement indicates that the Coach recognized he was an active participant in his mental health journey. Moreover, the repetitive use of words such as ‘confidence’ and ‘clarity’ across the various discussions with Coaches in Nairobi and Mombasa suggest a change in perceptions of self-identity. This and other examples conveyed that the MindSKILLZ program acted as a catalyst in fostering positive attitudes and resilience, thus supporting the mental health of the Coaches.

The words used by Coach Ann in Nairobi convey how she overcame emotional distress through her role as a MindSKILLZ Coach. She explained:

*Before joining the MindSKILLZ program, I was undergoing depression, even reaching to a point I felt like giving up. But through the MindSKILLZ program, I was able to identify my strengths, coping skills and realized it's okay not to be okay and I was able to overcome it.* (Coach in Nairobi).

The Coach’s use of “before” versus “but through” indicates a transformation which led to the Coach’s empowerment to overcome mental health challenges, and which helped her build resilience to face future adversity. Additionally, the Coach mentioned her recognition that, “It’s okay not to be okay,” demonstrating a change in her perspective on mental health.

b) **Improved self-esteem and academic performance of youth program participants**

The language used by Coaches in the FGDs further suggests that engagement in the MindSKILLZ program led to improved academic performance and self-esteem among youth program participants. This connotes how MindSKILLZ program interventions targeting improved mental health can have a ripple effect on academic outcomes among adolescent participants. Another narrative reinforced the observation that the interventions led to improved self-esteem of the adolescents. Speaking of one of their participants, Coach Juma in Mombasa said:

*Throughout the program, her self-esteem improved quite significantly to a point that she started talking about herself freely and interacting with the rest, MindSKILLZ is closely tied to performance of my students academically and notable changes in their social lives. It was impressive to note that majority of the students who underwent through the MindSKILLZ registered better performances. While speaking to some of their teachers, they expressed their satisfaction in how the students became actively involved in class discussions as compared to before* (Coach in Mombasa).

This narrative illustrates that the intervention was effective in improving the two unintended outcomes of increased self-esteem and improved academic performance, providing evidence to suggest program impact on the adolescents’ personal and academic achievements.

c) **Addressing structural barriers through advocacy**

The story of a MindSKILLZ participant, as described by Coach Buya in Nairobi, reflects power within (inner strength and self-belief) that helped a participant recognize their value and structural advocacy. Coach Buya narrated:

*[There was]* *a resilient young girl whose life took a positive turn, thanks to MindSKILLZ, a sport-based mental health program. After a session on seeking care and having supporters in life, that participant approached her Coach revealing the distressing reality of living with an abusive father who was beating her and her mother. The participants and her brother found refuge at their grandmother’s place, escaping the toxic environment back at home.* (Coach in Nairobi)

This discourse reflects on how the program was useful in helping young adolescents overcome a systemic issue—specifically, rampant domestic violence against them. The use of the word “resilient” by this Coach demonstrates strength shown by the program participants, while “whose life took a positive turn, thanks to MindSKILLZ” shows the transformative roles the program played for this youth participant. The narrative highlights structural barriers, particularly abuse and unsafe home environments, indicating the role of the program in facilitating safety and support for adolescents undergoing domestic violence.

**Discursive Practices (Meso Level)**

This section explores how discourses were produced, distributed, and consumed within a specific social context, mainly focusing on how these narratives were shaped by the intervention and how they were received by various audiences.

a) **Discourse Production**

The Coaches’ stories shared during the FGDs reveal how the MindSKILLZ program shaped the perspectives and behaviors of Coaches and participants alike. The program fostered an increased sense of empathy and emotional intelligence. During the FGD with Coaches in Mombasa, Coach Musyoka shared how the intervention changed the way he interacted with others, specifically referencing interactions with youth program participants:

*After going through the MindSKILLZ program, I now understand maybe someone can do something and it's not because they want to do it, but it's because even themselves, they're facing a certain challenge. When I understand that aspect… when one wrongs me, I just looked at them, I don't rant anymore. I just smile and then later on when the person is settled, I find out the problem and maybe there is also a challenge that person is undergoing. No wonder the person—maybe he is too angry having anger issues. Yeah, so nowadays I'm not judgmental. I just understand [the participants] and deal with them the way they are and sometimes I also help them deal with their mental health issues*. (FGD with Coaches in Mombasa)

This narrative shows how the program enabled some Coaches to reflect on their own biases. For example, Coach Musyoka in Mombasa shared, “…*Yeah, so nowadays I'm not judgmental. I just understand them and deal with them the way they are,”* indicating the adoption of an empathetic means to solve day-to-day issues which enhanced personal growth.

In her perspective, Coach Ruth in Nairobi shared the remarkable transformation of a participant, who after undergoing the MindSKILLZ program, internalized the teachings and used the various skills gained in regulating her emotions. She explained:

*The participant appreciated the techniques for managing stress and regulating her emotions, using practices such as feelings and emotions, awareness, power hand among other sessions. The interactive sessions provided a safe space for her to share her thoughts and feelings, fostering a sense of belonging within the practices.* (Coach in Nairobi)

However, some Coaches cited the pre- and post-surveys as being quite lengthy and burdensome for participants. Coach John in Nairobi explained, “…*Something that maybe I didn't like about MindSKILLZ – it's the pre-test and the post- tests. It's quite long for the participants.*” (FGD with Coaches in Nairobi).

b) **Discourse Distribution**

***Use of creative approaches to reach persons with disability***

Coaches adopted creativity while implementing the program to meet the needs of different participants who attended the MindSKILLZ sessions. Some Coaches revealed that they employed different creative efforts for participants with disability. Coach Musyoka narrated:

*[During] most MindSKILLZ games, participants are expected to stand, but my participants were in a wheelchair. So, they couldn't. We had to be creative enough to find ways in which they could participate in the games while sitting. So, instead of using the legs, we used the hands… but I loved the fact that as much as they were disabled, they were very active. And you could tell even when they came for graduation, I was shocked because some of them danced on the graduation day while in their wheelchairs, while holding their crutches… I loved the fact that we became creative—we even used some music because some of the games we couldn’t play for them to understand well.* (FGD with Coaches in Mombasa).

The use of these creative approaches demonstrates the ability of Coaches and the program to employ flexible transfer of knowledge to adolescents. While the narrative shows the ability of Coaches to adopt flexible ways in addressing some challenges, the FGD with Coaches in Mombasa noted a challenge in the distribution process. Coach Juma noted that there was need to have inclusive strategies to reach adolescents with disabilities. He stated challenges faced during text distribution activities and stressed the need for more inclusive strategies to reach adolescents with disabilities during such distribution activities as these efforts will accommodate all adolescents.

*They can teach us maybe how we can engage the disabled participants because these are these participants that could be mentally challenged, so we need to reach them as much as it is sport based. Maybe the MindSKILLZ team could come up with a way in which we can best engage the disabled participants.* (FGD with Coaches in Mombasa).

This challenge calls for a change in the design and delivery of the MindSKILLZ curriculum to ensure its effectiveness to all adolescents.

c) **Discourse Consumption**

***Enhancement of learning and transformative experience***

The MindSKILLZ program was utilized by different audiences, particularly adolescent participants and stakeholders, which suggests a wider reach potentially conveying broader societal impact. Coach Halima in Mombasa described the influence of MindSKILLZ on an adolescent as follows:

*Participation in the MindSKILLZ program has led to transformative behaviour in one adolescent girl, who previously struggled with using abusive language. Through engaging in the program's activities, she developed emotional sensitivity and self-awareness. As a result, she has grown into a respectful and considerate individual, demonstrating improved interpersonal relationships and a positive attitude toward others.* (Coach in Mombasa)

From the quote above, it is evident that the MindSKILLZ program reinforced the value in equipping adolescents with coping skills.

Additionally, the discussion with Coaches in Mombasa highlighted how the MindSKILLZ narrative was shaped by the broader social context in which the program operated. For example, Coach Aisha shared that the program enhanced learning and personal growth as she was able to reflect on her past experience and associate them with her younger self. She also appreciated the opportunity to interact with the community for the first time. Coach Aisha narrated:

*My journey in MindSKILLZ has been a journey of learning and learning and relearning. And the learning part was more of associating myself with the younger me. And it was a great experience also, MindSKILLZ gave me an opportunity to interact with the community, something that I've never done before, it's my first time.* (FGD with Coaches in Mombasa).

***Shaped ideological views***

The FGDs with Coaches also revealed how the program shaped ideological views by reinforcing the importance of emotional intelligence, which encouraged stakeholders to invest in such programs. Coach Hamisi in Mombasa recounted:

*The* *MindSKILLZ program was a turning point in my life. The program equipped me with tools to identify the triggers of my anger and develop strategies to manage it effectively. Through reflective practices, I learned the importance of emotional awareness and how to channel my feelings positively. Gradually, I began to experience inner peace and found healthier ways to express myself. I discovered that growth begins with acknowledging one’s struggles and taking intentional steps to address them.* (Coach in Mombasa)

The narrative connects emotional intelligence to broader developmental outcomes such as “*inner peace”* and “*healthier ways to express myself.”*

**Social Practice**

This section describes how Coaches’ discourse contributed to the maintenance of social power relations, ideologies, and institutional practices.

a) **Structural Power Imbalances**

Coach narratives highlight structural power imbalances in the mobilization and implementation processes for MindSKILLZ. Coaches and stakeholders played a successful role in the implementation of the program. They influenced the program’s acceptance, recruited participants, and created a conducive environment for learning. However, community health promoters (CHPs), who are typically critical in accessing the community, were not engaged in the program based on a decision made by LVCT Health. Coaches felt there was a need for inclusion of CHPs in the program implementation design for effective community engagement. As Coach Bahati stated, “*You can't just go to the community and start mobilizing for the children. You have to work with someone like a CHP, but you know, the slot for CHP was not included”* FGD with Coaches in Mombasa*.* Coaches were expected to mobilise participants but faced some challenges in the mobilization process. Coach Juma in Mombasa explained:

*Maybe I'm putting that across to be considered because mobilizing is a bit harder for us, but they* *[LVCT Health] should also include others, the gatekeepers like CHPs to help us in the mobilization…it'll help us to get more participants.* FGD with Coaches in Mombasa.

This indicates a mismatch between the institutional way of implementation and actual realities on the ground. While the institution may unintentionally reinforce power imbalances, the discourse calls for inclusion of CHPs to aid in the mobilization process.

b) **Social Power Relations**

Program documents such as the Coaches’ guide and MindSKILLZ magazine as well as the pre- and post- survey tools were originally written in English and then translated to formal Swahili language. This was after consultation with Youth Advisory Champions for Health (YACH) and LVCT Health involved in the design workshop. Coaches’ feedback on the translated materials revealed challenges particularly related to social power imbalances. For instance, Coach Halima in Mombasa observed “…*the Swahili translated version—it's the literate one and … the Swahili the community members are speaking, it's more different”* FGD with Coaches in Mombasa. Similar sentiments were shared by Coach Oluoch in Nairobi

*“I want to second R3 because… when it comes to curriculum, there are things like resilience, when you try to explain in the community, it's a little difficult. Terms such as, sympathy, empathy, how best can I explain it to someone in the community?”* FGD with Coaches in Nairobi.

This illustrates how formal language was used in the program documents over localized language, thus creating a gap in the program materials compared to the informal language used in the community. This gap placed the Coaches in a position of authority as they had to use a simplified language to pass messages to their participants. This reinforces power dynamics as the institution and the Coaches hold the knowledge of the formal language. In some instances, the Coaches felt that they might have simplified the information from the curriculum in a way that might have confused the participants or may not have gotten across the content effectively. Coach Halima in Mombasa shared:

*As much as you're going to translate …it, but it reaches a point that you feel like maybe you are off the point or maybe you've translated and you've confused them rather than giving them the information that you're supposed to give.* (FGD with Coaches in Mombasa)

In addition, the Coaches described some terminology specific to mental health concepts that needed to be translated to the participants for easier comprehension. Although the intention was to ensure clearer understanding, Coach Lulu in Mombasa observed that the translation carried unintended outcomes that in some cases brought confusion. The miscommunication may have contributed to participant dropout from the program, especially when some terminologies were equated with mental illness, as shared by Coach Lulu:

*When unpackaging the mental health program, when you mention issues of mental health, then you start speaking in Kiswahili because some of them, they don’t understand English. When you translate into Swahili, it becomes even more complicated. I remember even my last cohort, I lost almost 20 participants. I recruited 46, but by the time I was winding up on the program, it was about 23 because, after the introduction of mental health, the following day I would come and we asked why so and so didn’t come, and they would answer that they said they didn’t have mental health disorders, even some parents, and that's how we lost them. They say they don’t have a mental health disorder, when you address it as a mental health disorder, some of them understand it like, being mad, that you're not okay or just being mad.* (FGD with Coaches in Mombasa)

c) **Institutional Practices**

Some Coaches shared that certain MindSKILLZ sessions, concepts or practices assumed that all participants shared the same cultural experience. For example, Coaches explained that the use of standard guides assumed all groups were homogenous, yet the MindSKILLZ program was being implemented in entirely different and varying settings. The implementation was done in schools, community set-ups, and children’s homes. The variation in these set-ups indicates a variation in cultural experiences reflecting a challenge in LVCT health and GRS in standardizing approaches that enhance uniformity and inclusivity. The Coaches struggled to explain some terms from the written curriculum. For example, Coach Mercy in Nairobi highlighted, “*In the alcohol ‘facts and nonsense’ [activity], there's a whole statement about hangover... [The participants] have no idea [what a hangover is] …For the participant, they were really like, ‘What are you talking about?”* (FGD with Coaches in Nairobi).

One instance in which Coach Oluoch in Nairobi had to simplify the language points to institutional gaps to accommodate diverse and cultural backgrounds of the participants. Also, the assumption of globalized ideas, such as social media risk, alienated participants whose environments did not align with the broader global trends that are associated with social media. Coach Oluoch conveyed:

*There was another thing that I noted about [the ‘Take A stand’ activity] that says the number of social media followers that you have can define the how much support you get... This statement is not flowing the same in different settings. I've been in school, I've been in organizations, the children's home, and all of them get a different concept. Some don't even know what social media followers means…So, I felt that like we assumed…everybody knows what social media followers are, and it was not the case. Because even if I tried to explain what social media followers meant, okay, there are some that it was really simple for them to get that concept, but there were some which did not get into this environment that you are in.* (FGD with Coaches in Nairobi)

This represents a varying perspective against the existing community realities, thus, highlighting institutional discourse failure in the alignment of cultural contexts. The assumption upholds Westernized ideas of how social media define adolescence, which is not always the case in African communities.

**Discussion**

Using Fairclough’s CDA, this study set out to explore Coaches’ experiences as they participated in the implementation of MindSKILLZ program, including program impact on themselves and on the youth participants. According to the Coaches, the program fostered emotional intelligence and personal growth. They shared that the program content enabled them to also adopt attitudes to handle life challenges, with some developing skills such as self-awareness and stress management. These findings corroborate findings from previous studies on mental health, which have shown that mental health programs enhance an individual’s ability to maintain psychosocial state, thereby helping them to cope with daily activities (25). Furthermore, several Coaches also identified their own personal biases which previously influenced interactions with participants. Through the MindSKILLZ program, the Coaches improved their judgement and appreciation of individuals with diverse backgrounds and perspectives. These findings build on existing research which showed how interventions can improve and benefit not only the participants but also the facilitators. Results from a qualitative study conducted in Zambia highlighted how facilitators also experienced personal growth whilst implementing a youth-led intervention (26). In addition to improvements observed among Coaches, our findings also suggest that the MindSKILLZ program positively impacted on the youth participants. The findings suggest that the intervention enhanced adolescents’ self-esteem, which translated to better academic performance and increased class participation, highlighting perceived program effectiveness in fostering individual empowerment. These findings support the assertion of Nobre (2021) that mental health literacy at a younger age has a direct and positive impact on the overall wellbeing both in adolescents and adulthood. Through such initiatives, adolescents are able to acquire knowledge that shapes their behaviors and the attitudes that they carry in their future lives (27). Our findings support the literature that mental health programs build resilience and enhance emotions to build healthy relationships and positive sense of identity (28). Similarly, the opinion that adolescents improved academic performance was as a result of participating in MindSKILLZ aligns with prior research that has highlighted a correlation between an individual’s wellbeing and their performance in education. This signals the need for the integration of mental health education within the educational sector, especially in the Kenyan school system (29). Such integration would ensure that a program like MindSKILLZ positively impacts adolescents in school.

However, even with these successes of the MindSKILLZ program and Coaches adopting creative ways to implement the program especially with young people with disability. Coaches revealed implementation challenges, which emerged in relation to the Discursive Practice (Meso Level) and Social Practice (Macro Level) of Fairclough’s CDA. First, Coaches revealed challenges with engaging participants with disabilities. Most mental health programs have excluded people with disabilities, despite the fact that they face heightened risks of mental health challenges, often worsened by their disabilities and the stigma associated with them (30). Such inequities in health service provision among people with disability, particularly adolescents, is an indicator of scarce health intervention that targets this population especially in Low and Middle-Income Countries (LMICs) (31). Challenges to implementing such interventions include skills human resources required in the delivery of the interventions, cost of the interventions and the need for rigorous study designs (32). Even though Coaches employed creative approaches such as displaying Emoji cards to show feelings, and use of local songs to enhance participant comprehension, they also suggested the use of an inclusive curriculum to cater for persons with disability. Using findings from this study, GRS and LVCT Health have worked to make MindSKILLZ more inclusive for youth with disabilities. For example, a disability inclusion guidance document was created and distributed to all MindSKILLZ Coaches’, sharing practical implementation advice when working with participants with disabilities. This includes multiple adaptations for games and activities that can be utilized according to the abilities of participants in a particular group. Coaches received additional training on how to utilize the guidance document, and monitoring and evaluation of MindSKILLZ implementation with adolescents with disabilities is ongoing.

Structural power imbalances, social power relations, and institutional practices within the MindSKILLZ program impacted its implementation. The Kenya Community Health Policy 2020–2030 encourages community-based interventions to engage CHPs who reside in the local communities and speak the local language (33). However, for the MindSKILLZ interventions, CHPs who were more knowledgeable about the local geographies and communities were not involved in the recruitment of adolescent participants, yet the Coaches experienced challenges in reaching some participants who could have benefitted from the program. Studies emphasize the pivotal role CHPs play in providing access to communities especially in hard-to-reach areas (34). Involving CHPs in the recruitment process not only helps with engaging hard-to-reach populations but also increases the delivery of healthcare services to informal areas (35). It is evident that structural power imbalances influenced program implementation in that failure to involve CHPs in the implementation of project activities was a decision made by LVCT Health. To correct this, LVCT Health should consider involving CHPs in recruitment process in order to ensure inclusivity in program implementation.

Social power relations posed implementation challenges given how language issues generated unequal power dynamics between Coaches and youth participants. Despite a design workshop including youth representatives, LVCT Health and GRS staff, and key stakeholders in adolescent mental health in Kenya, Coaches highlighted where the language in the MindSKILLZ Coach’s Guide and Magazine fell short and was not easily understood by participants. Coaches’ way of translating and simplifying content for participants positioned them as figures of authority. Reliance on Coaches for simplifying and translating language amplified power imbalance, which at times resulted in misinterpretation and confusion. Global mental health initiatives have often been critiqued for lack of alignment with cultural context and conceptualization of mental health terminologies (36). A common challenge lies with mental health terminologies that are difficult to replicate in low-income settings. The tailoring of these terminologies to fit local context is both costly and difficult to replicate. In this pilot intervention, despite input from Kenyan youth and adults in the design phase, Coaches experienced difficulties conveying the formal Swahili translation of content to diverse groups of Kenyan youth. There is increasing recognition of the importance of culturally adapting intervention content to a specific population or setting (37). Our findings highlight the need to use culturally appropriate language as a key consideration in the delivery of an intervention in a new context (38). Understanding local mental health idioms and contextualization of such terminologies will enable recipients of mental health interventions to appreciate their meanings, and through this, they will be able to relate them to global mental health concepts (39). A co-creation workshop with the involvement of young people and local stakeholder to review translated materials can improve clarity and language contextualization.

**Study Strengths and Limitations**

This study applied sound qualitative design and utilized robust scientific methods which provided rich qualitative data outputs. Despite these strengths, the study notes a potential limitation on the use of CDA. The researchers' influences on data collection and translation of some focus group discussions from Swahili to English may have shaped the narratives which could have limited Coaches’ full expression of their experience in implementing the MindSKILLZ program. A reflexivity session was held to evaluate how subjectivity may have influenced the research process, though interpretation bias may be inevitable in this analysis.

**Conclusion**

Using Fairclough’s CDA to amplify Coaches’ voices by examining their personal stories highlights how discourses can reinforce and challenge social power imbalances and institutional practices. Coaches are imperative in the implementation of MindSKILLZ program, as they are considered change agents. Their discourse revealed the positive impact the program has had in fostering growth among participants and Coaches alike. Amplifying Coaches’ voices can facilitate a grassroots perspective on intervention delivery, which challenges power dynamics. The use of grassroots insights can aid in co-creating more equitable and effective mental health interventions. Findings from this study can further strengthen the refinement and future delivery of the MindSKILLZ mental health program and other mental health initiatives for youth in Kenya.

**Funding Statement**

This research was funded by Stavros Niarchos Foundation (SNF) through Grassroot Soccer, and was implemented jointly by Grassroot Soccer, LVCT Health, and the Departments of Health in Nairobi and Mombasa counties.

**References**

1.	Sequeira M, Singh S, Fernandes L, Gaikwad L, Gupta D, Chibanda D, et al. Adolescent Health Series: The status of adolescent mental health research, practice and policy in sub-Saharan Africa: A narrative review. Tropical Medicine and International Health. John Wiley and Sons Inc; 2022. p. 758–66. doi:10.1111/tmi.13802 PubMed PMID: 35906997.

2.	Africa S, Agyepong I, Sewankambo N, Binagwaho A, Coll-Seck AM, Corrah T, et al. Prof, MD; Ministry of Health, Dakar, Senegal 5. Prof, MD; MRC Gambia, Banjul, The Gambia 6. PhD; African Population and Health Research Center. National AIDS Control Council.

3.	Bago MH, Stephano EE, Kessy SA, Tibenderana JR, Godfrey Majengo V, Oguma ED, et al. Determinants of comprehensive HIV and AIDS knowledge among Tanzanian adolescent girls and young women: a multilevel application of Andersen’s behavioral model based on national survey. Front Public Health. 2025;13. doi:10.3389/fpubh.2025.1643422 PubMed PMID: 40994744.

4.	Nyundo A, Manu A, Regan M, Ismail A, Chukwu A, Dessie Y, et al. Factors associated with depressive symptoms and suicidal ideation and behaviours amongst sub-Saharan African adolescents aged 10-19 years: cross-sectional study. Tropical Medicine and International Health. 2020 Jan 1;25(1):54–69. doi:10.1111/tmi.13336 PubMed PMID: 31698526.

5.	Fleming T, Lewycka S, Kumah E, Mbinta J, Abubakar A, Mbithi G. Mental health interventions for adolescents in sub-Saharan Africa: A scoping review.

6.	WHO. Mental health of adolescents. 2024.

7.	Saade S, Lamarche AP, Khalaf T, Makke S, Legg A. What barriers could impede access to mental health services for children and adolescents in Africa? A scoping review. BMC Health Serv Res. 2023 Dec 1;23(1). doi:10.1186/s12913-023-09294-x PubMed PMID: 37024835.

8.	Seekles M, Twagira F, Alam A, Obasi A. Promotive and preventive interventions for adolescent mental health in Sub-Saharan Africa: a combined scoping and systematic review. BMJ Public Health. 2023 Dec;1(1):e000037. doi:10.1136/bmjph-2023-000037

9.	Steel J, Twagira F, Seekles ML, Obasi AI. Promotive, preventive, and treatment interventions for adolescent mental health in sub-Saharan Africa: A protocol for two scoping reviews including systematic analyses of intervention effectiveness. PLoS One. 2022 Dec 1;17(12 December). doi:10.1371/journal.pone.0279424 PubMed PMID: 36548366.

10.	Kumar M, Njuguna S, Amin N, Kanana S, Tele A, Karanja M, et al. Burden and risk factors of mental and substance use disorders among adolescents and young adults in Kenya: results from the Global Burden of Disease Study 2019. EClinicalMedicine. 2024 Jan 1;67. doi:10.1016/j.eclinm.2023.102328

11.	Lee DEZ, Nyauchi B, Kihiu M, Kimathi R, Okoth EA, Chazara A, et al. The MindSKILLZ sport-based mental health promotion intervention for adolescents in Kenya: a mixed methods pilot study. Front Public Health. 2026;14. doi:10.3389/fpubh.2026.1746268

12.	Memiah P, Wagner FA, Kimathi R, Anyango NI, Kiogora S, Waruinge S, et al. Voices from the Youth in Kenya Addressing Mental Health Gaps and Recommendations. Int J Environ Res Public Health. 2022 May 1;19(9). doi:10.3390/ijerph19095366 PubMed PMID: 35564760.

13.	Osborn TL, Venturo-Conerly KE, Wasil AR, Schleider JL, Weisz JR. Depression and Anxiety Symptoms, Social Support, and Demographic Factors Among Kenyan High School Students. J Child Fam Stud. 2020 May 1;29(5):1432–43. doi:10.1007/s10826-019-01646-8

14.	Mwangala PN, Kerubo A, Makandi M, Odhiambo R, Abubakar A. Prevalence and associated factors of mental and substance use problems among adults in Kenya: A community-based cross-sectional study. PLOS Global Public Health. 2025 Jun 1;5(6 June). doi:10.1371/journal.pgph.0004130

15.	LVCT Health. 2025. LVCT Health.

16.	GRS. 2025. Grassroot Soccer.

17.	Frontlineaids. MindSKILLZ – sports-based mental health programme for young people. 2024.

18.	Malla A, Boksa P, Joober R. The New Wave of Youth Mental Health Services: Time for Reflection and Caution. Canadian Journal of Psychiatry. 2021 Jul 1;66(7):616–20. doi:10.1177/0706743720984382 PubMed PMID: 33375848.

19.	Beji-Chauke R, Simms V, Abas M, Muzariri K, Mavhu W, Mangenah C, et al. A cluster-randomized trial of interventions for adolescent mental disorders in Zimbabwe. BMC Psychiatry. 2025 Dec 1;25(1). doi:10.1186/s12888-025-06755-x PubMed PMID: 40604530.

20.	McCabe E, Amarbayan M, Rabi S, Mendoza J, Naqvi SF, Thapa Bajgain K, et al. Youth engagement in mental health research: A systematic review. Health Expectations. John Wiley and Sons Inc; 2023. p. 30–50. doi:10.1111/hex.13650 PubMed PMID: 36385452.

21.	Janks H. Critical Discourse Analysis as a Research Tool. Johannesburg; 1996.

22.	Byansi W, Musyoka CM, Baidoo CE, Okumu M, Mutavi T, Mbwayo A, et al. Cumulative adverse childhood experiences and their association with depression and anxiety: a cross-sectional study of youth living in informal urban settings in Kenya. Front Psychiatry. 2025;16. doi:10.3389/fpsyt.2025.1641321

23.	Creswell JW, & PCN. Qualitative Inquiry and Research Design: Choosing Among Five Approaches . 4th ed. SAGE Publication; 2018.

24.	Olmos-Vega FM, Stalmeijer RE, Varpio L, Kahlke R. A practical guide to reflexivity in qualitative research: AMEE Guide No. 149. Med Teach. 2023;45(3):241–51. doi:10.1080/0142159X.2022.2057287

25.	Ahorsu DK, Sánchez Vidaña DI, Lipardo D, Shah PB, Cruz González P, Shende S, et al. Effect of a peer‐led intervention combining mental health promotion with coping‐strategy‐based workshops on mental health awareness, help‐seeking behavior, and wellbeing among university students in Hong Kong. Int J Ment Health Syst. 2021 Dec 1;15(1). doi:10.1186/s13033-020-00432-0

26.	Burke VM, Frimpong C, Miti S, Mwansa JK, Abrams EA, Merrill KG, et al. “It must start with me, so it started with me”: A qualitative study of Project YES! youth peer mentor implementing experiences supporting adolescents and young adults living with HIV in Ndola, Zambia. PLoS One. 2022 Feb 1;17(2 February). doi:10.1371/journal.pone.0261948 PubMed PMID: 35113861.

27.	Nobre J, Oliveira AP, Monteiro F, Sequeira C, Ferré-Grau C. Promotion of mental health literacy in adolescents: A scoping review. International Journal of Environmental Research and Public Health. MDPI; 2021. doi:10.3390/ijerph18189500 PubMed PMID: 34574427.

28.	Ungar M, Theron L. Resilience and mental health: how multisystemic processes contribute to positive outcomes. The lancet. Psychiatry. NLM (Medline); 2020. p. 441–8. doi:10.1016/S2215-0366(19)30434-1 PubMed PMID: 31806473.

29.	Hoover S, Bostic J. Schools as a vital component of the child and adolescent mental health system. Psychiatric Services. American Psychiatric Association; 2021. p. 37–48. doi:10.1176/APPI.PS.201900575 PubMed PMID: 33138711.

30.	Zuurmond M, Naami A, Adwan-Kamara L, Stephen C, Basnet S, Vanderick C, et al. Stigma reduction in a disability and mental health programme in Ghana: Lessons in participation. Afr J Disabil. 2025;14. doi:10.4102/ajod.v14i0.1508

31.	Hashemi G, Wickenden M, Bright T, Kuper H. Barriers to accessing primary healthcare services for people with disabilities in low and middle-income countries, a Meta-synthesis of qualitative studies. Disability and Rehabilitation. Taylor and Francis Ltd.; 2022. p. 1207–20. doi:10.1080/09638288.2020.1817984 PubMed PMID: 32956610.

32.	Martin Ginis KA, van der Ploeg HP, Foster C, Lai B, McBride CB, Ng K, et al. Participation of people living with disabilities in physical activity: a global perspective. The Lancet. Elsevier B.V.; 2021. p. 443–55. doi:10.1016/S0140-6736(21)01164-8 PubMed PMID: 34302764.

33.	Ministry of Health. Kenya_Palliative_Care_Policy_2021-2030_October_2021 [Internet]. Nairobi; 2021 Oct [cited 2025 Jan 17]. Available from: http://guidelines.health.go.ke:8000/media/Kenya_Palliative_Care_Policy_2021-2030_October_2021.pdf

34.	Lusambili AM, Nyanja N, Chabeda SV, Temmerman M, Nyaga L, Obure J, et al. Community health volunteers challenges and preferred income generating activities for sustainability: a qualitative case study of rural Kilifi, Kenya. BMC Health Serv Res. 2021 Jul 3;21(1):642. doi:10.1186/s12913-021-06693-w PubMed PMID: 34217281.

35.	Khatri RB, Endalamaw A, Erku D, Wolka E, Nigatu F, Zewdie A, et al. Enablers and barriers of community health programs for improved equity and universal coverage of primary health care services: A scoping review. BMC Primary Care. 2024 Dec 1;25(1). doi:10.1186/s12875-024-02629-5 PubMed PMID: 39472794.

36.	Sangraula M, Kohrt BA, Ghimire R, Shrestha P, Luitel NP, van’t Hof E, et al. Development of the mental health cultural adaptation and contextualization for implementation (mhCACI) procedure: a systematic framework to prepare evidence-based psychological interventions for scaling. Global Mental Health. 2021;8. doi:10.1017/gmh.2021.5

37.	Escoffery C LSEUHBEWRFMMP. A scoping study of frameworks for adapting public health evidence-based interventions. Transl Behav Med. 2019;1(9(1)):1–10.

38.	Bernal G, Bonilla J, Bellido C. Ecological Validity and Cultural Sensitivity for Outcome Research: Issues for the Cultural Adaptation and Development of Psychosocial Treatments with Hispanics. Joumal of Abnormal Child Psychology. 1995.

39.	Backe EL, Bosire EN, Kim AW, Mendenhall E. “Thinking Too Much”: A Systematic Review of the Idiom of Distress in Sub-Saharan Africa. Cult Med Psychiatry. 2021 Dec 1;45(4):655–82. doi:10.1007/s11013-020-09697-z PubMed PMID: 33387159.
