# Supplementary material for: Amplifying Coaches’ voices from the implementation of the MindSKILLZ youth mental health program in Kenya: A critical discourse analysis
Source: PLOS Glob Public Health. 2026 Sep 18;6(9):e0007336. doi: 10.1371/journal.pgph.0007336 (PMC13588515; doi:10.1371/journal.pgph.0007336)
Supplement: Supplementary file 6 [file pgph.0007336.s006.docx]

**Reviewer comments:**

Overall, I still believe that this is an interesting paper highlighting the important gap by focusing on the experiences of implementers (i.e., youth Coaches) rather than only participants. The paper illustrates how the perspectives of Coaches directly shape the delivery and effectiveness of youth mental health interventions.

**Materials and methods**

Previous comment:

Please provide more detail on the recruitment strategy described in paragraph 2, including how participants were identified and the sampling approach employed by the coaches.

Response: We used school registers and lists from the communities to randomly sample adolescent participants. Only those who provided parental consent and assent were enrolled into the program. Paragraph 3 under ‘Study Setting and Pilot Overview’

**New comment:**

Who was responsible for randomisation? Was it the coaches or the researchers? Which randomised sampling technique was used?

Characteristics of Study Participants – Thank you for adding the table and narrative; they help clarify the study population. To strengthen this section further, please consider reporting participants’ ages as a mean and standard deviation.

*“The adolescent participants were selected from the community and school set-ups after parental consent and assent were obtained.”* Clarify what is meant by “community and school set-ups.”

Does this refer to community centres, youth organisations, household-based recruitment, or another community-based setting?

Findings

*“Participation in the MindSKILLZ program has led to transformative behaviour in one adolescent girl, who previously struggled with using abusive language. Through engaging in the program's activities, she developed emotional sensitivity and self-awareness. As a result, she has grown into a respectful and considerate individual, demonstrating improved interpersonal relationships and a positive attitude toward others”*

From the quote above, it is evident that the MindSKILLZ program reinforced the value in

equipping adolescents with coping skills.

**Interpretation of the quotation:** Based on the quotation provided does not support that the program equipped adolescents with coping skills. Rather, it suggests that the programme strengthened emotional regulation and self-awareness, which are important components of coping.

**Discussion**

Paragraph 1 is too long; consider splitting it into two paragraphs for better clarity.
