# Supplementary material for: Amplifying Coaches’ voices from the implementation of the MindSKILLZ youth mental health program in Kenya: A critical discourse analysis
Source: PLOS Glob Public Health. 2026 Sep 18;6(9):e0007336. doi: 10.1371/journal.pgph.0007336 (PMC13588515; doi:10.1371/journal.pgph.0007336)
Supplement: Supplementary file 7 [file pgph.0007336.s008.docx]

| **Section** | **Reviewer’s comment** | **Author’s response** | **Additional notes** |
| --- | --- | --- | --- |
| **Reviewer 1** | | | |
| Abstract | Reviewer recommended the following corrections:   a) 5 replaced with five b) ‘to’ changed to ‘and’ | The authors accepted these changes | Page 1 |
| Introduction | Reviewer recommended the following corrections:   a) ‘drugs’ to ‘drug’ b) ‘abuse’ to ‘use’ c) Add ‘a’ between faces and severe d) Add ‘the’ before GRS e) Replace ‘on’ with ‘at’ | The authors accepted these changes. | Paragraphs 3,4 and 5 in pp2-3 |
| Materials and Methods | Reviewer recommended the following corrections:   a) Add ‘and’ between ‘wellbeing’ & ‘depressive symptoms’ b) Add ‘a’ before ‘monthly compensation’ | The authors accepted these changes. | Paragraphs 1 and 2 of Study Setting and Pilot Overview |
|  | The reviewer recommended conversion of Coaches compensation to US Dollars | The authors converted the amount at an exchange rate of 128.285 per Oanda on 7/8/2026 <https://www.oanda.com/currency-converter/en/> | The last sentence in paragraph 2 of Study Setting and Pilot Overview |
|  | Reviewer recommended addition of ‘likely’ before ‘influenced the research process’ | The authors accepted this change. | Paragraph 1 of the Reflexivity section |
|  | Reviewer recommended addition of ‘and/or’ before ‘were shaped by discourse’ | The authors accepted this change. | Bullet no 3 under Data Analyses |
| Findings | Reviewer recommended ‘illustrates’ instead of ‘illustrate’ | The authors accepted this change. | Paragraph 4 of p8 |
|  | Reviewer recommended addition of   a) Closing bracket b) Addition of ‘a’ before ‘participant’ | The authors accepted these changes. | Paragraph 1 of Addressing structural barriers through advocacy |
|  | Reviewer recommended addition of a comma after ‘MindSKILLZ program’ | The authors accepted this change. | Paragraph 3 under Discourse Production on p9 |
|  | Reviewer recommended ‘suggests’ instead of ‘suggest’ | The authors accepted this change. | Paragraph 1 under Discourse Consumption on p10 |
|  | Reviewer recommended the following:   a) deletion of ‘were’ before ‘translated to formal Swahili language’ b) replacing ‘clear’ with ‘clearer’ | The authors accepted these changes. | Paragraph 1 and 3 under Social Powe Relations on p12 and p13 |
|  | Reviewer recommended ‘alienated’ instead of ‘alienating’ | The authors accepted this change. | Paragraph 2 under Institutional Practices p13 |
| Discussion | The reviewer recommended the following:   a) Deletion of ‘s’ at the end of ‘programs’ b) Deletion of ‘on’ between ‘improved’ and ‘their judgement’ c) Replacement of ‘for’ with ‘of’ | The authors accepted these changes. | Paragraph 1 on p14 |
|  | The reviewer recommended the following:   a) ‘suggest’ instead of ‘suggested’ b) Addition of ‘on’ before ‘the youth participants’ c) Deletion of ‘the’ before ‘adolescents’ self-esteem’ d) ‘assertion’ instead of ‘arguments’ e) Deletion of ‘who suggests’ f) ‘younger’ instead of ‘young’ g) ‘and enhance’ instead of ‘enhancing’ | The authors accepted these changes. | Paragraph 2 on p14 and 15 |
|  | The reviewer recommended ‘resources’ in place of ‘resource’ | The authors accepted this change. | Paragraph 3 in p15 |
|  | The reviewer recommended deletion of space before start of sentence 3 | The authors accepted this change. | Paragraph 4 in p15 |
| Study Strengths and Limitations | The reviewer preferred ‘researchers’’ to replace of ‘researcher’s’ | The authors accepted this change. | Paragraph 1 in p16 |
| **Reviewer 2** | | | |
| General Comment | Overall, I still believe that this is an interesting paper highlighting the important gap by focusing on the experiences of implementers (i.e., youth Coaches) rather than only participants. The paper illustrates how the perspectives of Coaches directly shape the delivery and effectiveness of youth mental health interventions. | Thank you for this compliment. We strive to generate evidence from implementers with the aim of identifying challenges and solutions as well as strengthening their capacity towards improving their project performance |  |
| Materials and methods | Previous comment:  Please provide more detail on the recruitment strategy described in paragraph 2, including how participants were identified and the sampling approach employed by the coaches.  Response: We used school registers and lists from the communities to randomly sample adolescent participants. Only those who provided parental consent and assent were enrolled into the program. Paragraph 3 under ‘Study Setting and Pilot Overview’  **New comment:**  Who was responsible for randomisation? Was it the coaches or the researchers? Which randomised sampling technique was used? - | Randomisation was done by Coaches. This came after they had been trained on simple random sampling, which they subsequently used to recruit the study participants. | We have included additional information on the sampling procedure in Paragraph 3 in p14 |
|  | *“The adolescent participants were selected from the community and school set-ups after parental consent and assent were obtained.”* Clarify what is meant by “community and school set-ups.”  Does this refer to community centres, youth organisations, household-based recruitment, or another community-based setting? - We used schools and specific community boundaries as a sampling unit. | Within schools sampling was done as classroom level, while in communities sampling was done at household level |  |
| Findings | Characteristics of Study Participants – Thank you for adding the table and narrative; they help clarify the study population. To strengthen this section further, please consider reporting participants’ ages as a mean and standard deviation. | We had a mean age of 24.3 years. We have computed the standard deviation with an output of 1.86 years, and included this in the narrative. | Paragraph 1 of Characteristics of Study Participants in p6 |
|  | *“Participation in the MindSKILLZ program has led to transformative behaviour in one adolescent girl, who previously struggled with using abusive language. Through engaging in the program's activities, she developed emotional sensitivity and self-awareness. As a result, she has grown into a respectful and considerate individual, demonstrating improved interpersonal relationships and a positive attitude toward others”*  From the quote above, it is evident that the MindSKILLZ program reinforced the value in  equipping adolescents with coping skills  **Interpretation of the quotation:** Based on the quotation provided does not support that the program equipped adolescents with coping skills. Rather, it suggests that the programme strengthened emotional regulation and self-awareness, which are important components of coping. - | Thank you very much. We have revised the interpretation section based on this suggestion | Discourse Consumption in p11 |
| **Discussion** | Paragraph 1 is too long; consider splitting it into two paragraphs for better clarity. - | Thank you. We have split the paragraph into two to enhance clarity and coherency. | Pp14-15 |
