# Supplementary material for: Amplifying Coaches’ voices from the implementation of the MindSKILLZ youth mental health program in Kenya: A critical discourse analysis
Source: PLOS Glob Public Health. 2026 Sep 18;6(9):e0007336. doi: 10.1371/journal.pgph.0007336 (PMC13588515; doi:10.1371/journal.pgph.0007336)
Supplement: Peer review history

# Peer review: Decision Letter 0

**DOI:** 10.1371/journal.pgph.0007336.r001

**Authors:** Joel Francis

14 Apr 2026

PGPH-D-26-00596

Amplifying Coaches’ Voices from the Implementation of the MindSKILLZ Youth Mental Health Program in Kenya

PLOS Global Public Health

Dear Dr. Nyauchi,

Thank you for submitting your manuscript to PLOS Global Public Health. After careful consideration, we feel that it has merit but does not fully meet PLOS Global Public Health’s publication criteria as it currently stands. Therefore, we invite you to submit a revised version of the manuscript that addresses the points raised during the review process.

Please submit your revised manuscript by May 29 2026 11:59PM. If you will need more time than this to complete your revisions, please reply to this message or contact the journal office at globalpubhealth@plos.org. When you're ready to submit your revision, log on to https://www.editorialmanager.com/pgph/ and select the 'Submissions Needing Revision' folder to locate your manuscript file.

Please include the following items when submitting your revised manuscript:

- A letter that responds to each point raised by the editor and reviewer(s). You should upload this letter as a separate file labeled 'Response to Reviewers'.
- A marked-up copy of your manuscript that highlights changes made to the original version. You should upload this as a separate file labeled 'Revised Manuscript with Track Changes'.
- An unmarked version of your revised paper without tracked changes. You should upload this as a separate file labeled 'Manuscript'.

Guidelines for resubmitting your figure files are available below the reviewer comments at the end of this letter.

As the corresponding author, your ORCID iD is verified in the submission system and will appear in the published article. PLOS supports the use of ORCID, and we encourage all coauthors to register for an ORCID iD and use it as well. Please encourage your coauthors to verify their ORCID iD within the submission system before final acceptance, as unverified ORCID iDs will not appear in the published article. *Only*  the individual author can complete the verification step; PLOS staff *cannot*  verify ORCID iDs on behalf of authors.

We look forward to receiving your revised manuscript.

Kind regards,

Joel Msafiri Francis, MD, MS, PhD

Academic Editor

PLOS Global Public Health

Journal Requirements:

1. Please provide additional details regarding participant consent. In the ethics statement in the Methods and online submission information, please ensure that you have specified (1) whether consent was informed and (2) what type you obtained (for instance, written or verbal, and if verbal, how it was documented and witnessed). If your study included minors, state whether you obtained consent from parents or guardians. If the need for consent was waived by the ethics committee, please include this information.

If you are reporting a retrospective study of medical records or archived samples, please ensure that you have discussed whether all data were fully anonymized before you accessed them and/or whether the IRB or ethics committee waived the requirement for informed consent. If patients provided informed written consent to have data from their medical records used in research, please include this information.

1. We ask that a manuscript source file is provided at Revision. Please upload your manuscript file as a .doc, .docx, .rtf or .tex.

If the reviewer comments include a recommendation to cite specific previously published works, please review and evaluate these publications to determine whether they are relevant and should be cited. There is no requirement to cite these works unless the editor has indicated otherwise.

Additional Editor Comments (if provided):

[Note: HTML markup is below. Please do not edit.]

Reviewers' comments:

Reviewer's Responses to Questions

**Comments to the Author**

1. Does this manuscript meet PLOS Global Public Health’s publication criteria?

Reviewer #1: Yes

Reviewer #2: Yes

**********

2. Has the statistical analysis been performed appropriately and rigorously?

Reviewer #1: N/A

Reviewer #2: N/A

**********

3. Have the authors made all data underlying the findings in their manuscript fully available (please refer to the Data Availability Statement at the start of the manuscript PDF file)?

The PLOS Data policy

Reviewer #1: Yes

Reviewer #2: Yes

**********

4. Is the manuscript presented in an intelligible fashion and written in standard English?

Reviewer #1: No

Reviewer #2: Yes

**********

Reviewer #1: Overall

• The study rationale is unclear. Authors state: We were interested in understanding their experiences implementing a mental health intervention for young people during a pilot study, including any program impacts on Coaches themselves and on youth participants. Why was this done? To inform future programming?

• How does this approach compare to other approaches of using youth cadre to deliver mental health initiatives in this region e.g., Youth Friendship Bench. See: Beji-Chauke R, Simms V, Abas M et al. A cluster-randomized trial of interventions for adolescent mental disorders in Zimbabwe. BMC Psychiatry. 2025;25(1):673. doi: 10.1186/s12888-025-06755-x.

• The manuscript is marred by numerous typographical and grammatical errors. This affects the readability of this work. Careful proof reading and editing is needed.

• Title could include study type e.g. Amplifying Coaches’ Voices from the Implementation of the MindSKILLZ Youth Mental Health Program in Kenya: A Critical Discourse Analysis

Abstract

• Abstract should state study dates

Introduction

• HIV/AIDS remains a leading cause of death among adolescents in SSA – include citation.

• SSA also has the highest rate of adolescent’s pregnancies…- SSA also has the highest rate[s] of [adolescent]?…

• Evidence shows that interventions aimed at promoting mental wellbeing of adolescents and preventing mental health outcomes…

Check this - mental health outcomes can be either positive or negative. Positive mental health outcomes include for example, self-worth.

• In Kenya, many people attribute mental illness to spiritual or supernatural caused of mental illness – check this sentence.

• This lack of understanding contributes significantly to stigma surrounding mental health issues (7).

Unsure if this is a lack of understanding as mental illness could be due to many causes. Also, do authors intend to say mental illness or simply mental health problems. From which positionality are authors stating this? This is what people believe and this belief should be respected as opposed to being dismissed as ‘nonsense’.

• LVCT Health is a Kenyan non-governmental organization that implement [implements] health programs and scale [scales]?…

• By designing and implementing innovative health programs in the areas of HIV, sexual and reproductive health, maternal and child health, sexual violence prevention, mental health, and health systems strengthening(9) GRS is an adolescent health organization using the power of soccer to educate, inspire, and mobilize young people to live healthier lives across over 60 countries (10)(cite).

Is this one sentence? See (cite) at end of sentence.

Material, Methods and Approaches [Materials and Methods]

• Please include study guide as supplementary (S1) file

• and adolescents are predisposed to an environment that adversely affects their psychosocial health. Please say more about the environment.

• As part of the ITS design participant completed [participants]?

• All 30 Coaches were invited to participate in FGDs. Of these, 23 Coaches agreed to participate in 3 FGDs, with an average of 7 participants each.

Why did the other 7 refuse to participate?

• FGD [FGDs] together with Coaches’ personal stories

• They met regularly with two other researchers in a team of four to discuss the coding process, compare codes, and adjust approaches where needed to enhance consistency in the application of codes.

Were there any disagreements? If so, how were these resolved?

• …how this might have influenced the research process and the findings, [findings.]

Ethical Consideration[s]

• It also received approval by the [from the]

Results

Text (Micro Level) This analysis focused on the textual and linguistic features of Coaches’ statements, all of which represented success stories relating to the program’s impact and implementation.

This is repetitive.

• participants living with disability [participants with disability]

• adolescents living with disabilities [adolescents with disabilities]

• institution way [institutional]?

• Coaches suggested that the language translated in the program documents and pre and post survey that were written in English Coaches feedback on program documents and pre and post survey revealed challenges particularly related to social power imbalances.

Check this.

• set -ups [set-ups] also spelt setups

• approaches that enhances [enhance]

• This conflict with community realities highlighting institutional discourse failure in the alignment of cultural contexts.

Discussion

• A qualitative study conducted in Zambia results highlights how facilitators also experienced personal growth whilst implementing a youth led intervention (21).

Check this.

• In addition to improvements observed among Coaches our findings also revealed [suggested]?

• especially with young people living with disability [especially with young people with disability]

• have excluded people living with disabilities [people with disabilities]

• Health inequities for children is an indicator of scarce health intervention[s]…Lower Middle-Income Countries [Low to Middle-Income Countries]?

• Studies emphasizes [emphasize]

• According to the Kenya Community Health Policy 2020–2030, CHPs are selected within the localities that they reside and who are able to speak the local language (25).

Check this.

• Limitations section could also focus on strengths e.g., Study strengths and limitations.

Reviewer #2: Overall, I believe that this is an interesting paper highlighting the important gap by focusing on the experiences of implementers (i.e., youth Coaches) rather than only participants. The paper illustrates how the perspectives of Coaches directly shape the delivery and effectiveness of youth mental health interventions.

Introduction

• ‘poor reproductive and sexual health outcomes, and troubled parent/child relationships (1).’ Consider rewriting it to ‘Poor sexual and reproductive health outcomes’

• Are these rates referring to prevalence in SSA or different countries? Please make this clear

• ‘ref’ – please kindly add the reference

• The inclusion of Kenya would benefit from clearer integration into the narrative. It may be helpful to introduce the focus on Kenya more explicitly and provide a rationale for its inclusion in relation to the study. This would strengthen the argument regarding the implementation of the intervention and enhance the overall relevance of the study within the Kenyan context.

• reads like it was forgotten and included at the last minute. Please find a way to introduce Kenya and explain why focusing on Kenya as a whole is relevant to the study. It would support the argument that is being made about the implementation of the intervention and the relevance of the study in Kenya.

• You can also highlight the mental health scope in Kenya before discussing the lack of support. This will strengthen the argument you are making.

• If data is available, you can describe the ratio of individuals with mental health conditions to available health personnel in Kenya. This would help to contextualise the capacity of the health system.

• If possible, please spell out LVCT in full at first mention before using the acronym throughout the paper

• Add a full stop after reference 9

• After reference 10, you wrote ‘cite’ – not sure if you forgot to remove this or wanted to include another citation. Please update accordingly.

Methodology

• Provide references in paragraph 1 in the last two sentences.

• What is GRS? Please spell out the acronym to ensure clarity.

• Briefly explain the ‘necessary knowledge and skills' that the coaches require to deliver the sessions.

• Please provide more detail on the recruitment strategy described in paragraph 2, including how participants were identified and the sampling approach employed by the coaches.

• Please briefly describe the content of the ‘pre’ and ‘post’ surveys mentioned in paragraph 2, including the key domains or topics covered.

• Please clarify what is meant by a ‘participant in need’. Additionally, indicate whether any follow-up procedures were conducted to assess whether participants accessed the recommended services, and if so, which services were utilised (paragraph 3)

• Were the coaches compensated?

Findings

Kindly describe the population (how many coaches, age, education status, etc)

b) Improved self-esteem and academic performance of the youth program participants.

- Based on the quote provided, I don’t believe it captures what you described as ‘This observation connotes how self-esteem can have a ripple effect on academic outcomes.’ I can see the improved self-esteem and academic performance, but from the provided quotes, I can’t see how one informs the other or the link that you infer in the paragraph after the quote on self-esteem.

- Throughout the program, her self-esteem improved quite significantly to a point that she started talking about herself freely and interacting with the rest

o Was this interaction observed in class, too or in the program?

b) Social Power Relations

- Rewrite the first sentence; it is not clear.

- If possible, please provide examples of the specific terminology being referred to. Additionally, it may be useful to clarify whether the challenge was primarily related to the terminology itself or to how these concepts were explained during translation. Based on the quote provided, it appears that some participants may have interpreted the sessions as being targeted at individuals with mental health disorders, rather than as an opportunity to learn about mental health more broadly. Clarifying this distinction could strengthen the interpretation of participant dropout.

Discussion

- Rewrite the 6th sentence that speaks about the results from Zambia.

- ‘Most mental health programs have excluded people living with disabilities, despite the fact that they face heightened risks of mental health challenges, often worsened by their disabilities and the stigma associated with them.’ - Reference this statement.

- Rewrite the first part of the sentence following the discussion about health inequities in children.

- For the last sentence in paragraph 3, maybe you can provide examples of what the coaches employed.

- If possible, maybe describe why a decision was made to exclude the CHPs from recruiting.

- ‘According to the Kenya Community Health Policy 2020–2030, CHPs are selected within the localities that they reside and who are able to speak the local language (25)’ This sentence feels a bit misplaced; maybe move it up or elaborate more in terms of the importance of CHPs according to the health policy to support your argument.

**********

what does this mean?). If published, this will include your full peer review and any attached files.

**Do you want your identity to be public for this peer review?**  If you choose “no”, your identity will remain anonymous but your review may still be made public.

For information about this choice, including consent withdrawal, please see our Privacy Policy

Reviewer #1: No

Reviewer #2: No

**********

[NOTE: If reviewer comments were submitted as an attachment file, they will be attached to this email and accessible via the submission site. Please log into your account, locate the manuscript record, and check for the action link "View Attachments". If this link does not appear, there are no attachment files.]

# Author comment: Author response to Decision Letter 1

**DOI:** 10.1371/journal.pgph.0007336.r002

2 Jun 2026

Attachment Submitted filename: Response to Reviewers.docx

# Peer review: Decision Letter 1

**DOI:** 10.1371/journal.pgph.0007336.r003

**Authors:** Joel Francis

19 Jul 2026

PGPH-D-26-00596R1

Amplifying Coaches’ Voices from the Implementation of the MindSKILLZ Youth Mental Health Program in Kenya: A Critical Discourse Analysis

PLOS Global Public Health

Dear Dr. Nyauchi,

Thank you for submitting your manuscript to PLOS Global Public Health. After careful consideration, we feel that it has merit but does not fully meet PLOS Global Public Health’s publication criteria as it currently stands. Therefore, we invite you to submit a revised version of the manuscript that addresses the points raised during the review process.

Please submit your revised manuscript by Aug 18 2026 11:59PM. If you will need more time than this to complete your revisions, please reply to this message or contact the journal office at globalpubhealth@plos.org. When you're ready to submit your revision, log on to https://www.editorialmanager.com/pgph/ and select the 'Submissions Needing Revision' folder to locate your manuscript file.

Please include the following items when submitting your revised manuscript:

- A letter that responds to each point raised by the editor and reviewer(s). You should upload this letter as a separate file labeled 'Response to Reviewers'.
- A marked-up copy of your manuscript that highlights changes made to the original version. You should upload this as a separate file labeled 'Revised Manuscript with Track Changes'.
- An unmarked version of your revised paper without tracked changes. You should upload this as a separate file labeled 'Manuscript'.

Guidelines for resubmitting your figure files are available below the reviewer comments at the end of this letter.

As the corresponding author, your ORCID iD is verified in the submission system and will appear in the published article. PLOS supports the use of ORCID, and we encourage all coauthors to register for an ORCID iD and use it as well. Please encourage your coauthors to verify their ORCID iD within the submission system before final acceptance, as unverified ORCID iDs will not appear in the published article. *Only*  the individual author can complete the verification step; PLOS staff *cannot*  verify ORCID iDs on behalf of authors.

We look forward to receiving your revised manuscript.

Kind regards,

Joel Msafiri Francis, MD, MS, PhD

Academic Editor

PLOS Global Public Health

**Journal Requirements:**

If the reviewer comments include a recommendation to cite specific previously published works, please review and evaluate these publications to determine whether they are relevant and should be cited. There is no requirement to cite these works unless the editor has indicated otherwise.

Please review your reference list to ensure that it is complete and correct. If you have cited papers that have been retracted, please include the rationale for doing so in the manuscript text, or remove these references and replace them with relevant current references. Any changes to the reference list should be mentioned in the rebuttal letter that accompanies your revised manuscript. If you need to cite a retracted article, indicate the article’s retracted status in the References list and also include a citation and full reference for the retraction notice.

**Additional Editor Comments (if provided):**

[Note: HTML markup is below. Please do not edit.]

Reviewers' comments:

Reviewer's Responses to Questions

**Comments to the Author**

Reviewer #1: All comments have been addressed

Reviewer #2: All comments have been addressed

**********

publication criteria?

Reviewer #1: Yes

Reviewer #2: Yes

**********

3. Has the statistical analysis been performed appropriately and rigorously?

Reviewer #1: N/A

Reviewer #2: N/A

**********

4. Have the authors made all data underlying the findings in their manuscript fully available (please refer to the Data Availability Statement at the start of the manuscript PDF file)?

The PLOS Data policy

Reviewer #1: No

Reviewer #2: Yes

**********

5. Is the manuscript presented in an intelligible fashion and written in standard English?

Reviewer #1: Yes

Reviewer #2: Yes

**********

**Reviewer #1** : See attached.

**Reviewer #2:**  Reviewer comments:

Overall, I still believe that this is an interesting paper highlighting the important gap by focusing on the experiences of implementers (i.e., youth Coaches) rather than only participants. The paper illustrates how the perspectives of Coaches directly shape the delivery and effectiveness of youth mental health interventions.

Materials and methods

Previous comment:

Please provide more detail on the recruitment strategy described in paragraph 2, including how participants were identified and the sampling approach employed by the coaches.

Response: We used school registers and lists from the communities to randomly sample adolescent participants. Only those who provided parental consent and assent were enrolled into the program. Paragraph 3 under ‘Study Setting and Pilot Overview’

New comment:

• Who was responsible for randomisation? Was it the coaches or the researchers? Which randomised sampling technique was used?

Characteristics of Study Participants

Thank you for adding the table and narrative; they help clarify the study population. To strengthen this section further, please consider reporting participants’ ages as a mean and standard deviation.

“The adolescent participants were selected from the community and school set-ups after parental consent and assent were obtained.” Clarify what is meant by “community and school set-ups.”

• Does this refer to community centres, youth organisations, household-based recruitment, or another community-based setting?

Findings

“Participation in the MindSKILLZ program has led to transformative behaviour in one adolescent girl, who previously struggled with using abusive language. Through engaging in the program's activities, she developed emotional sensitivity and self-awareness. As a result, she has grown into a respectful and considerate individual, demonstrating improved interpersonal relationships and a positive attitude toward others”

From the quote above, it is evident that the MindSKILLZ program reinforced the value in equipping adolescents with coping skills.

Comment:

• Interpretation of the quotation: Based on the quotation provided does not support that the program equipped adolescents with coping skills. Rather, it suggests that the programme strengthened emotional regulation and self-awareness, which are important components of coping.

Discussion

Paragraph 1 is too long; consider splitting it into two paragraphs for better clarity.

**********

what does this mean?). If published, this will include your full peer review and any attached files.

**Do you want your identity to be public for this peer review?**  If you choose “no”, your identity will remain anonymous but your review may still be made public.

For information about this choice, including consent withdrawal, please see our Privacy Policy

Reviewer #1: No

Reviewer #2: No

**********

[NOTE: If reviewer comments were submitted as an attachment file, they will be attached to this email and accessible via the submission site. Please log into your account, locate the manuscript record, and check for the action link "View Attachments". If this link does not appear, there are no attachment files.]

**Figure Resubmissions:**

Attachment Submitted filename: Manuscript wm.docx

Attachment Submitted filename: renamed_b7287.docx

# Author comment: Author response to Decision Letter 2

**DOI:** 10.1371/journal.pgph.0007336.r004

17 Aug 2026

Attachment Submitted filename: Response to Reviewers-13Aug2026.docx

# Editor report: Decision Letter 2

**DOI:** 10.1371/journal.pgph.0007336.r005

**Authors:** Joel Francis

1 Sep 2026

Amplifying Coaches’ Voices from the Implementation of the MindSKILLZ Youth Mental Health Program in Kenya: A Critical Discourse Analysis

PGPH-D-26-00596R2

Dear Mr Nyauchi,

We are pleased to inform you that your manuscript 'Amplifying Coaches’ Voices from the Implementation of the MindSKILLZ Youth Mental Health Program in Kenya: A Critical Discourse Analysis' has been provisionally accepted for publication in PLOS Global Public Health.

Before your manuscript can be formally accepted you will need to complete some formatting changes, which you will receive in a follow up email. A member of our team will be in touch with a set of requests.

Please note that your manuscript will not be scheduled for publication until you have made the required changes, so a swift response is appreciated.

IMPORTANT: The editorial review process is now complete. PLOS will only permit corrections to spelling, formatting or significant scientific errors from this point onwards. Requests for major changes, or any which affect the scientific understanding of your work, will cause delays to the publication date of your manuscript.

If your institution or institutions have a press office, please notify them about your upcoming paper to help maximize its impact. If they'll be preparing press materials, please inform our press team as soon as possible -- no later than 48 hours after receiving the formal acceptance. Your manuscript will remain under strict press embargo until 2 pm Eastern Time on the date of publication. For more information, please contact globalpubhealth@plos.org.

Thank you again for supporting Open Access publishing; we are looking forward to publishing your work in PLOS Global Public Health.

Best regards,

Joel Msafiri Francis, MD, MS, PhD

Academic Editor

PLOS Global Public Health

***********************************************************

Reviewer Comments (if any, and for reference):
